# In vivo coupling of tau pathology and cortical thinning in Alzheimer's disease

**DOI:** 10.1016/j.dadm.2018.08.005

**Published:** 2018-09-17

**Authors:** Elijah Mak, Richard A.I. Bethlehem, Rafael Romero-Garcia, Simon Cervenka, Timothy Rittman, Silvy Gabel, Ajenthan Surendranathan, Richard W. Bevan-Jones, Luca Passamonti, Patricia Vázquez Rodríguez, Li Su, Robert Arnold, Guy B. Williams, Young T. Hong, Tim D. Fryer, Franklin I. Aigbirhio, James B. Rowe, John T. O'Brien

**Affiliations:** a Department of Psychiatry, University of Cambridge, Cambridge, UK; b Department of Clinical Neurosciences, University of Cambridge, Cambridge, UK; c Department of Clinical Neuroscience, Centre for Psychiatry Research, Karolinska Institutet and Stockholm County Council, Stockholm, Sweden; d Laboratory for Cognitive Neurology, University of Leuven, Leuven, Belgium; e China-UK Centre for Cognition and Ageing Research, Southwest University, Chongqing, China; f Wolfson Brain Imaging Centre, University of Cambridge, Cambridge, UK; g Medical Research Council, and Brain Sciences Unit, Cambridge, UK; h Behavioural and Clinical Neuroscience Institute, University of Cambridge, Cambridge, UK

**Keywords:** Alzheimer's disease, Tau, Amyloid, Positron emission tomography, Atrophy, Cortical thickness, MRI

## Abstract

**Introduction:**

The deposition of neurofibrillary tangles in neurodegenerative disorders is associated with neuronal loss on autopsy; however, their in vivo associations with atrophy across the continuum of Alzheimer's disease (AD) remain unclear.

**Methods:**

We estimated cortical thickness, tau ([^18^F]-AV-1451), and amyloid β (Aβ) status ([^11^C]-PiB) in 47 subjects who were stratified into Aβ− (14 healthy controls and six mild cognitive impairment–Aβ−) and Aβ+ (14 mild cognitive impairment–Aβ+ and 13 AD) groups.

**Results:**

Compared with the Aβ− group, tau was increased in widespread regions whereas cortical thinning was restricted to the temporal cortices. Increased tau binding was associated with cortical thinning in each Aβ group. Locally, regional tau was associated with temporoparietal atrophy.

**Discussion:**

These findings position tau as a promising therapeutic target. Further studies are needed to elucidate the casual relationships between tau pathology and trajectories of atrophy in AD.

## 1 Background

The prevailing disease model of Alzheimer's disease (AD) implicates amyloidosis as the initiating pathologic event, followed by a cascade involving aggregation of neurofibrillary tangles (NFTs), early synaptic dysfunction, downstream progressive cerebral atrophy, and ultimately clinical and functional decline [1]. However, evidence from postmortem and positron emission tomography (PET) studies has not been able to demonstrate strong associations of amyloid β (Aβ) with neuronal loss or disease severity in AD [2], [3]. In contrast, NFTs accumulate in tandem with neuronal loss, disease progression, and show strong correlations with clinical phenotypes [4], [5], [6], findings which have since been corroborated by cerebrospinal fluid evidence implicating tau as a key substrate of brain atrophy across various neurodegenerative conditions [7], [8], [9], [10].

The advent of PET radiotracers that bind to hyperphosphorylated paired helical filaments of aggregated tau has permitted us to characterize the in vivo spatial distribution of tau burden, and how it relates to other pathologic processes in the AD cascade. To these ends, the neuropathologic staging of tau has been consistently recapitulated across research groups: tau pathology is localized in the medial temporal lobe among cognitively normal elderly adults before extending to the posterior parietal cortices in mild cognitive impairment (MCI) and AD [11], [12], [13], [14], [15]. One of the recurrent themes in the tau imaging literature concerns the striking overlap of increased [^18^F]-AV-1451 binding with brain regions that comprise the AD “cortical signature” of atrophy [16], suggesting a close coupling between tau and downstream neurodegeneration. To date, only a few studies have delineated these relationships in cognitively normal elderly [17], [18], [19] and small samples of patients with AD [20]. It also remains unclear if and to what extent does Aβ levels modify the relationships between tau and brain atrophy.

The objective of our study was to elucidate the relationships between tau pathology and brain atrophy across individuals varying degrees of Aβ burden. We used a multimodal paradigm that included [^11^C]-PiB PET for Aβ classification in MCI individuals, [^18^F]-AV-1451 PET for quantification of tau pathology, and T1-magnetization-prepared rapid gradient echo (MPRAGE) for estimation of cortical thickness. Individuals with mild AD and [^11^C]-PiB + MCIs were treated as a single group, because these individuals represent a continuum from prodromal to early AD. We further examined the impact of tau on brain atrophy in another group comprising cognitively normal elderly and [^11^C]-PiB − MCI individuals, thereby enabling us to inquire whether the influence of tau on brain atrophy may be influenced by existing amyloid burden. First, we compared the spatial distributions of tau burden and cortical thickness between both Aβ subgroups. Second, we tested the hypothesis that the global topography of tau closely overlaps with cortical atrophy. Third, we directly mapped local burden of tau pathology onto regional cortical thickness. Finally, the distributed patterns of tau-associated atrophy were investigated using a seed-based approach, with the inferior temporal tau selected as a proxy of early tau propagation.

## 2 Methods

### 2.1 Participants

As part of the Neuroinflammation in Memory and Related Other Disorders study [21], 20 MCI and 13 AD subjects were recruited from cognitive disorder clinics in neurology, old age psychiatry, and related services at Cambridge University Hospital and other Trusts within the region. MCI was defined as (1) Mini-Mental State Examination (MMSE) >24; (2) not demented but with memory impairment beyond that expected for age and education, which does not meet the criteria for probable AD dementia and is not explained by another diagnosis [22]. Probable AD was diagnosed according to the National Institute on Aging-Alzheimer's Association diagnostic guidelines [23]. Fourteen healthy control subjects were recruited from spouses of subjects and from volunteers. They were defined as subjects with MMSE scores >26 and with an absence of (1) regular memory complaints; (2) symptoms suggestive of dementia; and (3) unstable or significant medical illnesses. All research participants underwent a detailed clinical and neuropsychological assessment as previously described [15].

### 2.2 Image acquisition

Participants underwent T1-weighted magnetic resonance imaging (MRI) using an MPRAGE sequence (repetition time = 2300 ms, echo time = 2.98 ms, field of view = 240 mm, flip angle = 9°) on a Siemens 3 T Tim Trio or Verio (Siemens Healthcare, Erlangen, Germany). PET examinations were performed on the GE Advance or GE Discovery 960, with the tau radioligand [^18^F]-AV-1451 (Avid Radiopharmaceuticals, Philadelphia, PA, USA). A 15-minute ^68^Ge/^68^Ga rotating rod transmission scan was used for attenuation correction on the Advance, which was replaced by a low-dose computed tomography scan on the Discovery 690. The PET examination protocols were the same for both scanners: 550 MBq [^11^C]-PiB injection followed by acquisition of static emission data from 40 to 70 minute after an injection; and collection of 90-minute dynamic data after a 370 MBq [^18^F]-AV-1451 injection. Each emission frame was reconstructed using the Project Missing Data three-dimensional filtered back projection algorithm into a 128 × 128 matrix 30 cm transaxial field of view, with a transaxial Hann filter cutoff at the Nyquist frequency. Corrections were applied for randoms, dead time, normalization, scatter, attenuation, and sensitivity. In addition, subjects with MCI underwent [^11^C]-PiB PET imaging to quantify the density of fibrillar Aβ deposits for classification of Aβ (PiB cortical standardized uptake value ratio [SUVR] >1.5) [24].

### 2.3 Processing of structural MRI and PET data

#### 2.3.1 Structural MRI

The T1-MPRAGE data were processed with FreeSurfer v6 to obtain cortical thickness measurements in 34 regions of interest (ROIs) per hemisphere, based on the Desikan-Killiany parcellation scheme [25], [26]. Briefly, for each T1-MPRAGE data, the pial and white matter surfaces were generated and the cortical thickness was measured as the distance between the boundaries of pial and white matter surfaces. Visual inspection was carried while blinded to group diagnosis and corrections were performed to ensure accurate skull stripping and reconstruction of white matter and pial surfaces, and one AD subject was excluded as a result.

#### 2.3.2 [^18^F]-AV-1451

The [^18^F]-AV-1451 emission image series were aligned across the frames to correct for head motion during data acquisition with statistical parametric mapping (SPM8). The realigned dynamic frames were coregistered to the T1-MPRAGE. The data were corrected for partial volume effects with the symmetric geometric transfer matrix in PetSurfer, following previously adopted procedures in a growing number of multimodal PET and MRI studies [17], [27]. Using the gray matter cerebellum as the reference region, kinetic modeling was performed using the two-stage Multilinear Reference Tissue Model [28] within the PetSurfer pipeline to derive partial volume corrected nondisplaceable binding potential (BP_ND_) values for each ROI [29].

#### 2.3.3 [^11^C]-PiB

[^11^C]-PiB data were quantified using an SUVR with the superior cerebellar gray matter as the reference region. The [^11^C]-PiB SUVR data were similarly subjected to the geometric transfer matrix technique for partial volume correction and treated as a dichotomous variable for Aβ classification. MCI subjects were classified as Aβ+ if the averaged cortical [^11^C]-PiB SUVR was >1.5 [24]. This classification resulted in 14 Aβ+ and six Aβ− MCI subjects.

### 2.4 Statistical analyses

All statistical analyses were performed in MATLAB 2017A and *R*. First, linear regressions were performed to adjust the imaging data for age, gender, and scan interval between structural MRI and PET assessments, consistent with our previous methodology [30]. The specific analyses catering to the main objectives of the study are described as follows: (1) Student's *t* tests were used to compare regional tau burden and cortical thickness between the Aβ− (healthy control subjects and MCI-Aβ−) and Aβ+ (MCI-Aβ+ and AD) groups and corrected for multiple comparisons with Benjamini-Hochberg false discovery rate (FDR; adjusted *P* < .05). (2) To examine the spatial overlap between tau and cortical thickness, we used mixed effects models to evaluate the inter-regional associations between both imaging modalities across the cortex. Specifically, cortical thickness was assigned as the dependent variable, with [^18^F]-AV-1451 BP_ND_ as a fixed factor, allowing for random intercepts across subjects and cortical lobes. A second reduced model was derived by omitting the fixed effects of [^18^F]-AV-1451 BP_ND_ from the original model. Likelihood ratio tests were used to infer statistical significance by comparing the fit between the full and reduced models [31]. (3) To delineate the topography of local relationships between tau and cortical thickness, we pursued an unbiased approach and investigated correlations between the adjusted [^18^F]-AV-1451 BP_ND_ and cortical thickness data within the same ROI. One-way analysis of covariance was performed with the Aβ+ and Aβ− groups as a factor and cortical thickness as a covariate to investigate potential interactions of Aβ status on tau-associated cortical thinning. (4) To investigate the local-to-distributed influence of tau pathology, we selected the inferior temporal cortex as a proxy measure of early tau seeding and assessed its correlations with cortical thickness ROIs. Two AD subjects were excluded from the statistical analyses as they were outliers on [^18^F]-AV-1451 BP_ND_ data (Grubb's test) and were inflating many of the regional correlations between tau burden and cortical thickness.

## 3 Results

### 3.1 Demographics of study sample

Participant clinical and demographic characteristics are shown in Table 1. Although there were no significant differences between both Aβ groups in terms of age, gender, and education, the Aβ+ group was significantly more impaired on the MMSE and underwent a longer scan interval between MRI and PET imaging.

**Table 1:** Sample characteristics

Variables	Healthy control subjects and MCI-Aβ−	MCI-Aβ+ and AD	*P* value
*N*	20	24	
Age (y)	70 ± 8.8	74.0 ± 8.0	.1∗
Male:female	12:8	14:10	.9†
MMSE	28.8 ± 2.1	26.1 ± 1.9	<.001‡
Education (y)	14.4 ± 2.8	13 ± 3.2	.1‡
Scan interval (d)	72.1 ± 81.5	204.8 ± 169.6	.002‡

Abbreviations: Aβ, amyloid β; AD, Alzheimer's disease; MCI, mild cognitive impairment; MMSE, Mini-Mental State Examination, MRI, magnetic resonance imaging.

∗ *t* Test.

† χ^2^ Test.

‡ Mann-Whitney rank sum.

### 3.2 Global and regional comparisons of cortical thickness and tau accumulation

**Fig. 1:**
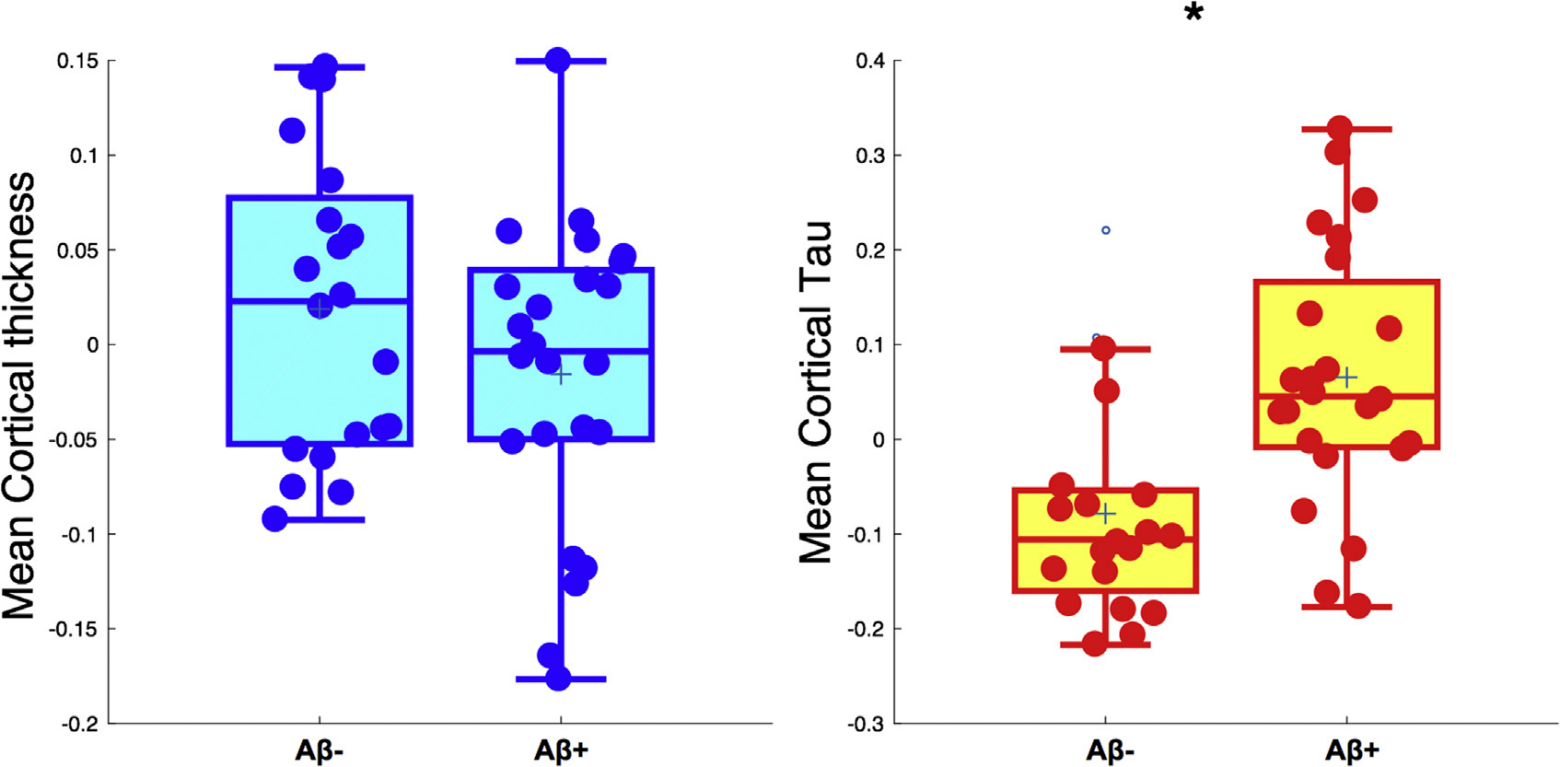
Between-group comparisons of mean cortical thickness and [^18^F]-AV-1451 burden. Student's *t* tests revealed no significant differences in mean cortical thickness between Aβ groups, although tau accumulation was significantly increased in the Aβ+ group (*P* < .001). Abbreviation: Aβ, amyloid β.

**Fig. 2:**
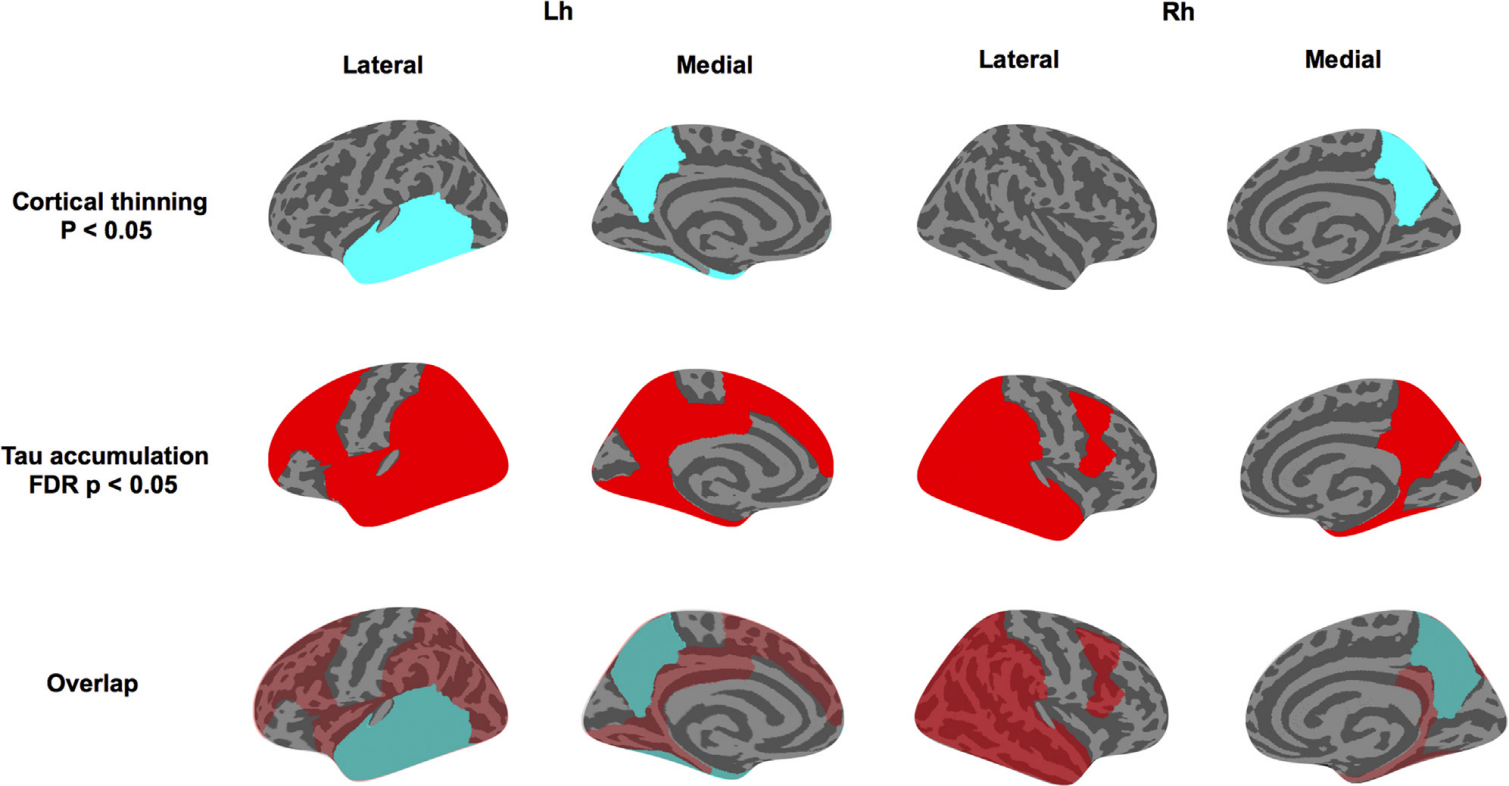
Group comparisons of regional cortical thickness and tau accumulation between both Aβ subgroups. (Top and middle rows) Relative to the Aβ− group, the magnitude and spatial extent of tau accumulation (red, FDR *P* < .05) were in excess of trend-level cortical thinning (cyan, *P* < .05). (Bottom row) The spatial overlap in the distributions of cortical thinning and tau accumulation is visually apparent when the contrast maps are superimposed on each other. Abbreviations: Aβ, amyloid β; FDR, false discovery rate.

Although there were no significant differences in mean cortical thickness (*P* = .2), mean cortical tau burden was significantly increased in the Aβ+ group relative to the Aβ− group (*P* < .001) (Fig. 1). Next, we compared the regional cortical thickness and [^18^F]-AV-1451 binding between both groups. Relative to the Aβ− group, the Aβ+ group exhibited a trend-level pattern of cortical thinning that was largely restricted to the temporal cortices and bilateral precuneus (*P* < .05; Fig. 2, top row). However, these differences did not survive FDR correction across the 68 ROIs. In contrast, significantly increased [^18^F]-AV-1451 binding was observed in widespread regions, predominantly spanning the temporoparietal cortices in the Aβ+ group (FDR corrected, *P* < .05; Fig. 2, middle row). Topographically, the trend-level pattern of cortical thinning was embedded within a wider extent of tau accumulation (Fig. 2, bottom row).

### 3.3 Topographical relationship between tau accumulation and cortical thickness

**Fig. 3:**
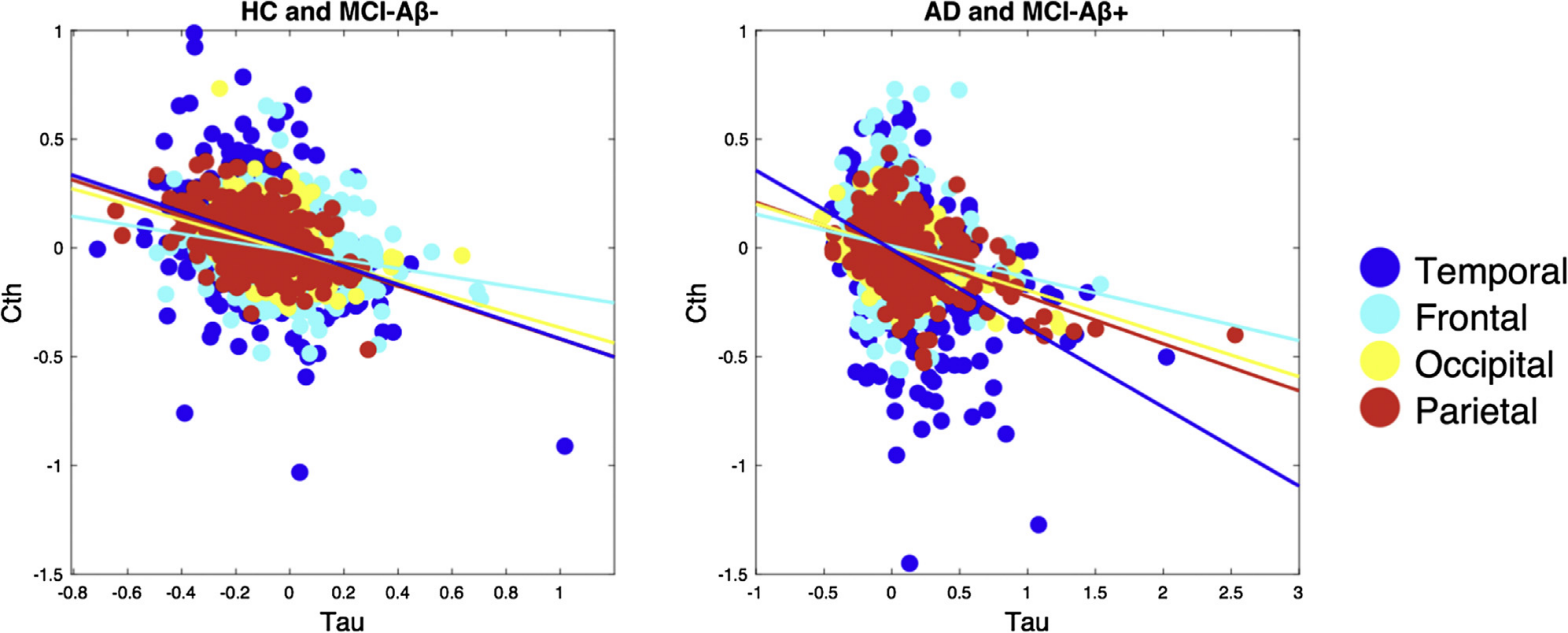
The cortical topography of tau pathology overlaps with reduced cortical thickness in both Aβ subgroups. Mixed effect models indicated significant and negative associations between [18F]-AV-1451 binding and cortical thickness in both groups (left: Aβ−: β = −0.5, SE = 0.03, *T* = −14.6, *P* < .001; right: Aβ+: β = −0.3, SE = 0.02, *T* = −14.8; *P* < .001). The scatterplots depict individual data points of the adjusted [^18^F]-AV-1451 BP_ND_ and cortical thickness data across the subjects (i.e., data adjusted for age, gender, and scan interval days between PET and MRI). Abbreviations: Aβ, amyloid β; BP_ND_, nondisplaceable binding potential; HC, healthy controls; MCI, mild cognitive impairment; MRI, magnetic resonance imaging; PET, positron emission tomography; SE, standard error.

Mixed effect models indicated significant and negative associations between tau burden and cortical thickness irrespective of Aβ grouping (Aβ−: β = −0.5, standard error = 0.03, *T* = −14.6; Aβ+: β = −0.3, standard error = 0.02, *T* = −14.8). These topographical associations are illustrated in the scatterplots of Fig. 3. In addition, we evaluated the robustness of these relationships within each cortical lobe. Intralobar associations from the mixed effect models are reported in Supplementary Fig. 1 and Supplementary Table 1, showing consistent, significant associations of tau pathology with cortical thinning across the lobes.

### 3.4 Local associations of tau pathology with cortical thickness

**Fig. 4:**
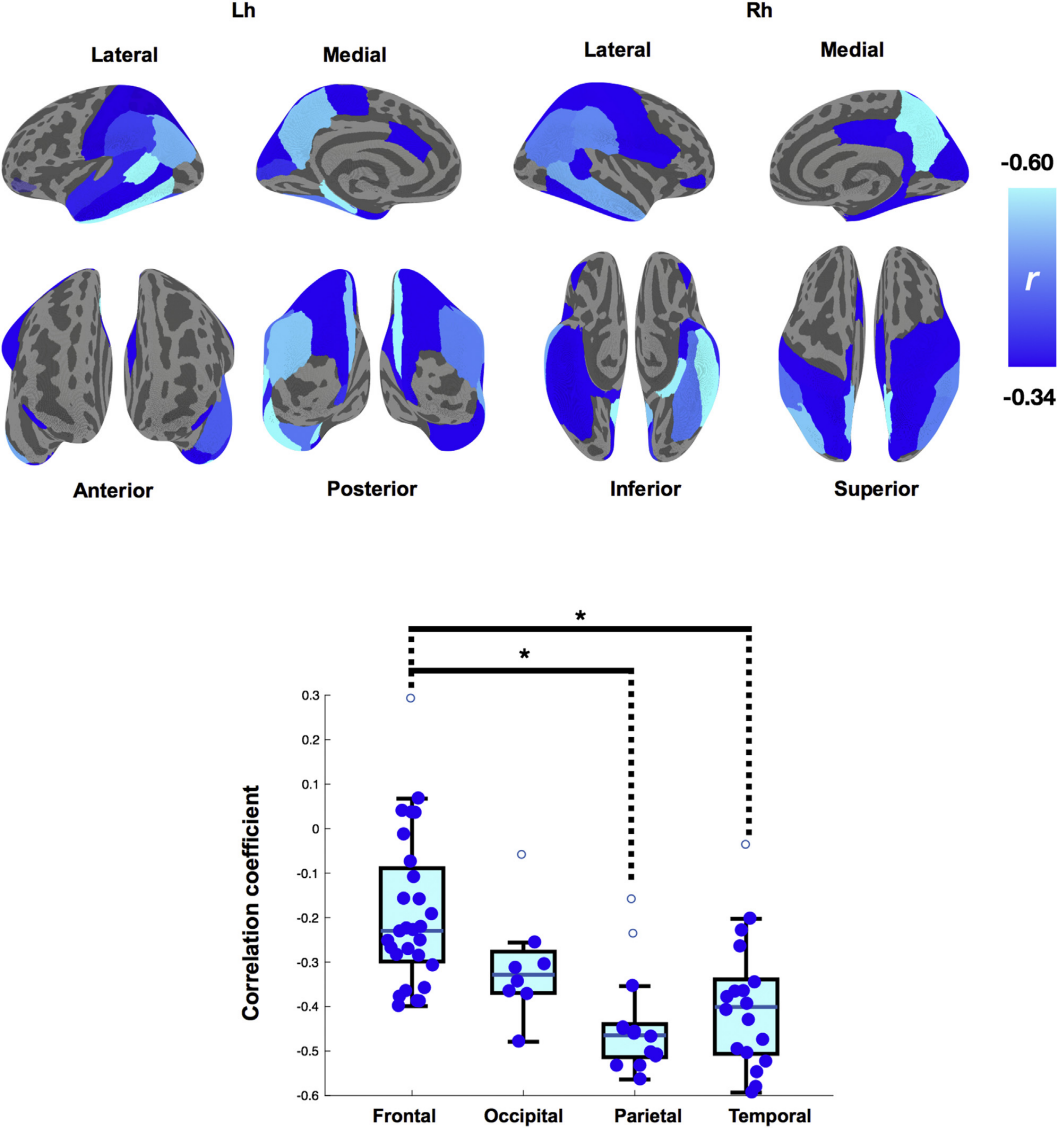
The topography of tau-associated brain atrophy. (Top row) Significant local correlations between tau and cortical thickness are overlaid on the cortical surface as parcellated by the Desikan-Killiany atlas (FDR *P* < .05, data adjusted for age, gender, and scan interval between PET and MRI). The color gradient represents the strength of the negative correlations, increasing in magnitude from dark blue to cyan. (Bottom row) Box plots of correlation coefficients across the major cortical lobes. The degree of local associations was significantly stronger in the temporal and parietal lobes compared with the frontal lobe (Post hoc Tukey-HSD, *P* < .05). Abbreviations: FDR, false discovery rate; MRI, magnetic resonance imaging; PET, positron emission tomography.

Although previous analyses examined the degree of global and lobar overlap between tau burden and cortical thinning, here we delineated the extent to which the regional intensity of tau burden is associated with cortical thinning within the same ROI. We observed strong local associations between tau burden and cortical thinning in widespread regions. The most robust associations that retained significance after Benjamini-Hochberg FDR correction for multiple comparisons were predominantly in the temporoparietal cortices. The spatial profile of these local associations is represented as a heat map on the Desikan-Killiany template, where the color gradient depicts the strength of the local correlations (i.e., increasing in magnitude from blue to cyan) (Fig. 4, top row). The regional scatterplots are also reported in Supplementary Fig 2. Visually, the heat map suggested that tau-associated cortical thinning followed a posterior bias across the cortex. This was subsequently confirmed by a significant main effect of lobes in our analysis of variance comparisons of the correlational coefficients (*F* [3, 67] = 11.7, *P* < .001). Post hoc Tukey–Honest Significant Difference tests revealed significantly stronger local associations in both the temporal and parietal lobes relative to the frontal lobe (Fig. 4, bottom row). Furthermore, one-way analysis of covariance was performed with the Aβ+ and Aβ− groups as a factor and cortical thickness as a covariate, although we did not find any significant interaction of Aβ status on the relationship between tau and cortical thickness.

### 3.5 Local and distributed patterns of cortical thinning associated with inferior temporal tau

**Fig. 5:**
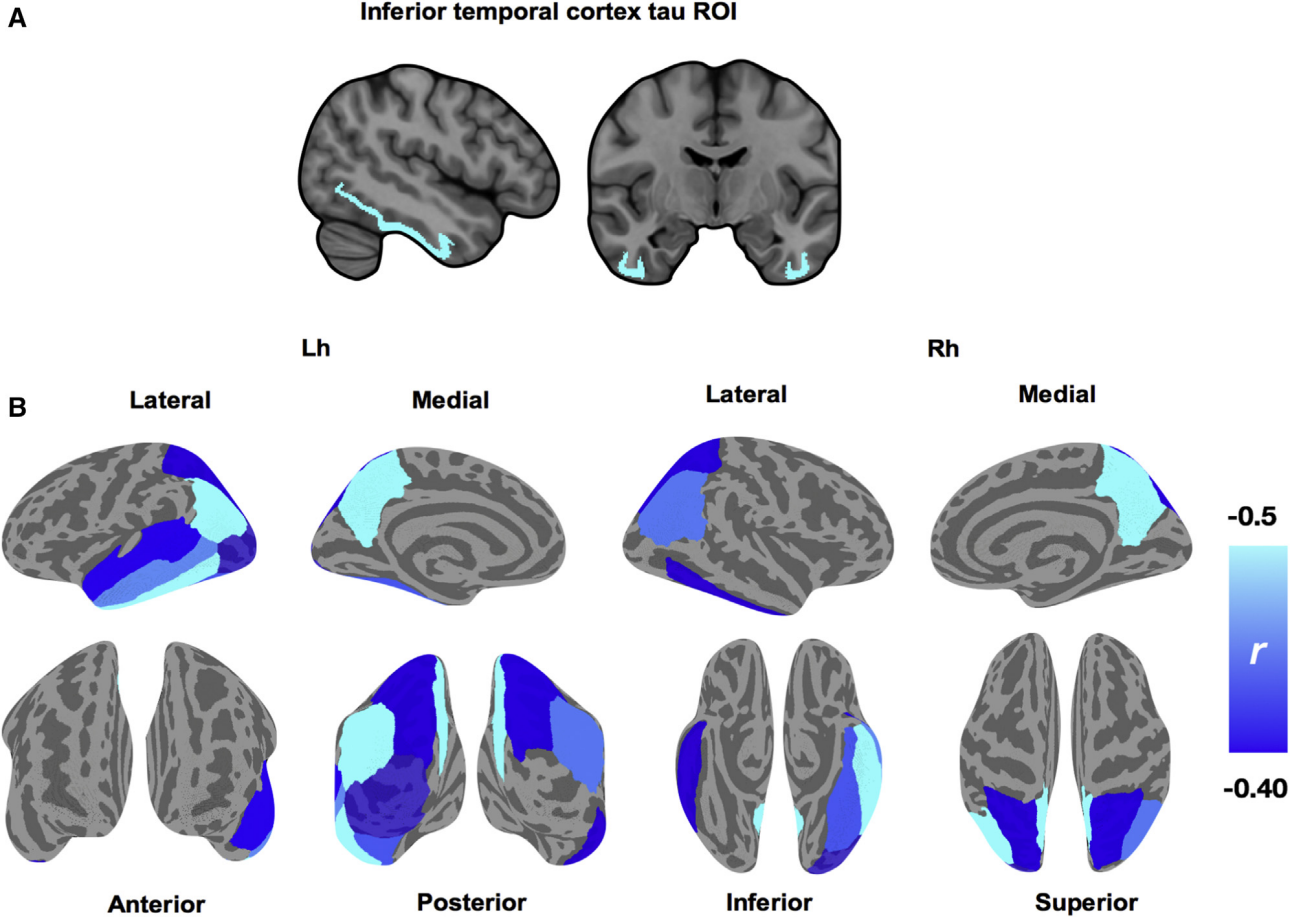
Delineating the local and distributed associations of tau in inferior temporal cortex and cortical thinning. (A) Mean PET signal was extracted from bilateral inferior temporal cortex for each subject and their associations with cortical thickness were assessed with Spearman correlations due to non-normality of the inferior temporal tau ROI. (B) Significant local correlations surviving FDR correction are overlaid on the cortical surface, as parcellated by the Desikan-Killiany atlas. The color gradient represents the strength of the negative correlations, increasing in magnitude from dark blue to cyan. Abbreviations: FDR, false discovery rate; PET, positron emission tomography.

In addition to local atrophy, inferior temporal tau burden was significantly associated with cortical thinning in multiple nearby regions within the temporal lobe (left temporal banks, left fusiform gyrus, left middle temporal cortex, left superior temporal cortex) and distant regions including the bilateral inferior parietal cortex, left lateral occipital cortex, bilateral precuneus, and right superior parietal cortex (Fig. 5 and Supplementary Fig. 4; FDR *P* < .05). There were no significant interactions of Aβ group.

## 4 Discussion

Determining the in vivo relationships between tau pathology and other neurodegenerative processes is essential for the evaluation of early biomarkers and to facilitate the development of therapeutic candidates in AD. Our findings collectively demonstrated that tau pathology, measured in vivo with [^18^F]-AV-1451 PET, is strongly associated with cortical thinning. In addition, we demonstrated that the phenomenon of tau-associated atrophy exists irrespective of amyloid burden. Broadly, these findings suggest that the impact of tau pathology on brain atrophy may be underway even at subthreshold accumulation of Aβ, raising the possibility that early anti-tau interventions may have greater therapeutic potential than anti-Aβ, especially early in the course of disease.

Recent PET imaging studies have demonstrated close relationships of tau aggregation with Aβ burden [33], [34], [35] and hypometabolism [32]. In addition to corroborating previous findings in populations of cognitively normal elderly and smaller samples of patients with different AD variants (*n* = 6) [17], [20], our study also confirmed the large body of neuropathologic evidence implicating NFTs as a precursor of downstream neuronal loss in AD. However, previous studies have relied on case-control samples or did include groups with varying degrees of amyloid burden. As such, our findings extended the literature by demonstrating a tight coupling between tau and atrophy that may not be contingent on existing severity of Aβ burden.

Despite being a necessary condition of AD, the precise involvement of Aβ underlying disease progression or brain atrophy has been tenuous (see [36] for a systematic review). For instance, it remains uncertain if—and to what extent—tauopathy in the absence of Aβ can perpetuate the neurodegenerative cascade that ultimately leads to clinical and functional impairment. Our mixed effect analyses revealed prominent associations between increased tau burden and cortical thinning, which were surprisingly also found among individuals classified in the Aβ− group (Fig. 3). Furthermore, we did not detect a significant interaction of Aβ levels on the local-to-local associations between tau burden and atrophy (Supplementary Fig. 2). Together, both lines of evidence may be interpreted as evidence against the hypothesis of a dose-dependent relationship between tau-associated atrophy and severity of Aβ levels. These findings are broadly consistent with recent data showing Aβ-independent relationship between tau and hypometabolism in a large sample of cognitively normal elderly [37]. Indeed, the practical implications of these observations are manifold. First, the presence of tau-related atrophy in individuals with minimal Aβ may reflect subtle neurodegeneration that coexists with primary age-related tauopathy. The ubiquity of NFTs is well documented in the brains of the older population, even in the absence of Aβ plaques, and may be associated with mild or diffuse cortical atrophy [38]. Second, these findings could be taken to support the growing recognition that clearance of Aβ pathology alone is insufficient as a treatment approach, with the corollary that anti-tau interventions may have more therapeutic potential in the early phases of AD. Indeed, in contrast to the prevailing theory that tau hyperphosphorylation is secondary to the build up of Aβ, other groups have argued that tau pathology is a necessary precursor for Aβ−induced neurotoxicity [39], thereby highlighting the potential of tau-targeting therapies to have beneficial impact on both pathologies [40]. Taken together, these findings position tau pathology as an important and early therapeutic target, even in preclinical AD.

After demonstrating the spatial concordance of tau and cortical thinning across the cortex as well as within each lobe, we delineated the cortical landscape of colocalized tau and atrophy. As hypothesized, we found significant local relationships that were predominantly in the inferior temporal and parietal cortices, retaining statistical significance even at a relatively stringent FDR-adjusted threshold (Fig. 4, top row). The posterior bias of the local associations, confirmed by our analysis of variance comparisons of the intralobar correlational coefficients (i.e., temporoparietal lobes > frontal lobe; Fig. 4, bottom row), is in keeping with the Braak staging of tau propagation where tau first originates in the medial temporal lobe before spreading to posterior cortices along neural pathways [41]. Rather intriguingly, the topography of tau-associated atrophy in this study is highly reminiscent of the cortical signature of AD, a set of brain regions that are highly susceptible to undergo atrophy in patients with established AD [16]. Extrapolating the concept of ischemic penumbra to our observation, it is conceivable that peak regions showing the strongest tau-atrophy correlations may form a “neurodegenerative penumbra” that subsequently serves as the pathologic scaffold from which atrophy ultimately emerges in a pattern akin to the cortical signature of AD. Such a model would be consistent with evidence from transgenic mice that Aβ plaques in situ have a penumbra of soluble Aβ oligomers in which the loss of synaptic density decreases at further distances from the plaque edge [42]. In other words, these tau-related associations may reflect the initial processes affecting structural morphology, and thus represent a pattern of disease propagation in AD. Longitudinal studies will be necessary to disentangle the temporal sequence of these events. If our hypothesis is borne out in prospective and longitudinal studies, it would provide compelling evidence for a mechanistic bridge between tau, a cardinal pathologic substrate of AD, and cortical atrophy—the common end point of neurodegeneration.

In addition to tau-related local atrophy, distal neurodegeneration may also be plausible through means of connectional diaschisis. Using the inferior temporal cortex as a proxy of tau burden within a seed-based framework, we showed that its associations with atrophy extended locally to encompass adjacent temporal cortices and the bilateral precuneus (Fig. 5). These results also confirm previous studies, in cognitively normal individuals, that implicated inferior temporal tau with diffuse patterns of atrophy and Aβ in temporoparietal cortices [17], [18]. Interpreted from a network-mapping perspective [43], these findings are in keeping with a role of the inferior temporal cortex as a “gateway region” for disease propagation in AD. To elucidate the diaschisistic underpinnings of tau-related neurodegeneration in AD, one key area of interest will be in discerning the remote consequences of local tau pathology on the connectomic architecture in AD.

Finally, through the between-group comparisons of tau and cortical thickness, we endeavored to indirectly probe the spatiotemporal relationships between tau and atrophy with complementary lines of evidence. Relative to Aβ− group, the Aβ+ group showed a pattern of increased tau accumulation that was more widespread than atrophy, which only followed a restricted trend of cortical thinning in the temporal cortices. Interestingly, the atrophic sites—temporal cortex and precuneus—were embedded and surrounded by regions of significant tau accumulation, raising the possibility that these regions may represent the “epicenters” of AD. This notion is in accord with previous evidence demonstrating early vulnerability of the precuneus and the medial temporal lobe in AD [44]. Taken together, the disproportionate increase in tau accumulation relative to cortical thinning also confers primacy to NFTs as an upstream event relative to atrophy.

Several important caveats should be noted. Given the sample size, our findings will benefit from further replication in larger samples, although it is assuring that our findings retained statistical significance even after stringent FDR correction and adjustment for important covariates, such as age, gender, and scan interval durations between PET and MRI assessments. In the absence of longitudinal data, our inferences regarding the spatiotemporal relationships between tau and atrophy are limited by the assumption that cross-sectional measurements are indices reflecting the summed pathologic accumulation over time. However, these processes may or may not follow a linear trajectory and accrual of these measures does not necessarily reflect the duration of their presence.

## 5 Conclusions

The findings in this report serve to triangulate observations from postmortem and cerebrospinal fluid studies and provide in vivo evidence that tau aggregation is tightly associated with both the spatial profile and severity of brain atrophy. Of note, we further showed the consistency of these relationships across groups with varying degrees of Aβ pathology, suggesting that tau pathology should be recognized as an early therapeutic target in preclinical AD. Locally, the distributions of tau-associated cortical thinning are strikingly reminiscent of the cortical signature of AD and may indicate early vulnerability to the neurotoxicity of AD-related pathologies. Ultimately, although this study is the first to comprehensively delineate the topography of tau-associated atrophy, we stress that prospective longitudinal studies with larger samples are necessary to replicate our findings.

**Research in Context**

1. Systematic review: We recently published a systematic review of tau positron emission tomography imaging studies in 2017, and further reviewed the literature (i.e., PubMed). There are very few investigations into the associations of tau positron emission tomography with brain atrophy, and samples in previous studies mainly involved cognitively elderly cohorts or smaller case series with AD. These studies have been cited.
2. Interpretation: Consistent with the aforementioned evidence in normal aging cohorts, our findings suggest that tau pathology is strongly associated with stereotypical patterns of atrophy that recapitulated the cortical signature of AD.
3. Future directions: Longitudinal designs are necessary to replicate these findings in larger prospective cohorts comprising individuals across the disease spectrum of the AD.
