# Supplementary material for: In vivo coupling of tau pathology and cortical thinning in Alzheimer's disease
Source: Alzheimers Dement (Amst). 2018 Sep 17;10:678–87. doi: 10.1016/j.dadm.2018.08.005 (PMC6222030; doi:10.1016/j.dadm.2018.08.005)
Supplement: Supplementary Material [file mmc1.docx]

IN VIVO COUPLING OF TAU PATHOLOGY AND CORTICAL THINNING IN NORMAL AGEING AND ALZHEIMER’S DISEASE

**SUPPLEMENTARTY MATERIALS**


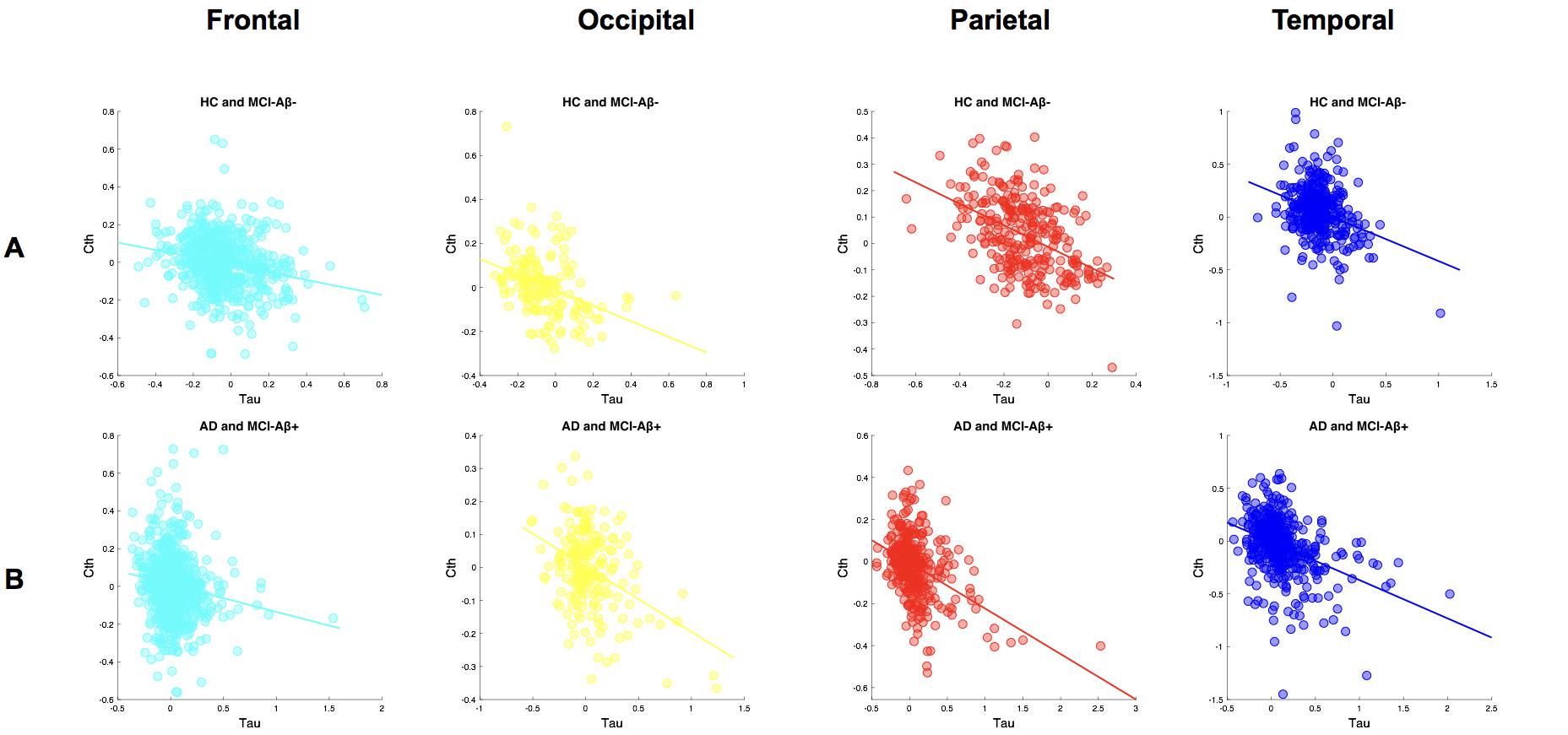


**Supplementary Figure 1. Intra-lobar relationships between tau accumulation and cortical thinning.** Mixed effect models, accounting for repeated measurements per subject, indicated consistent and significant associations between [^18^F]-AV151 binding and cortical thinning within each lobe and across Aβ groups (see Supplementary Table 1 for raw values). The scatterplots depict individual data points of the [^18^F]-AV1451 BP_ND_ and cortical thickness across the subjects (i.e. data adjusted for age, gender, and scan-interval days between PET and MRI).

| **Lobe** | **Aβ -** | | | | **Aβ +** | | | |
| --- | --- | --- | --- | --- | --- | --- | --- | --- |
|  | **β** | **SE** | ***T*** | **p** | **β** | **SE** | ***T*** | **p** |
| **Frontal** | -0.4 | 0.05 | -7.9 | ** | -0.1 | 0.04 | -3.1 | * |
| **Occipital** | -0.4 | 0.06 | -6.9 | ** | -0.2 | 0.03 | -8 | ** |
| **Parietal** | -0.5 | 0.05 | -8.6 | ** | -0.3 | 0.03 | -9.8 | ** |
| **Temporal** | -0.5 | 0.07 | -6.8 | ** | -0.3 | 0.05 | -7.4 | ** |

**Supplementary Table 1. Intra-lobar mixed effect modelling of the relationships between [^18^F]-AV1451 binding and cortical thickness, stratified by Aβ subgroup**. Abbreviations: β = Estimate, SE = Standard error, *T* = T value, ** p < 0.001, * p < 0.05. In all models, statistical significance of the mixed effect models was inferred using chi-square comparison of log-likelihood ratios against null models.

*
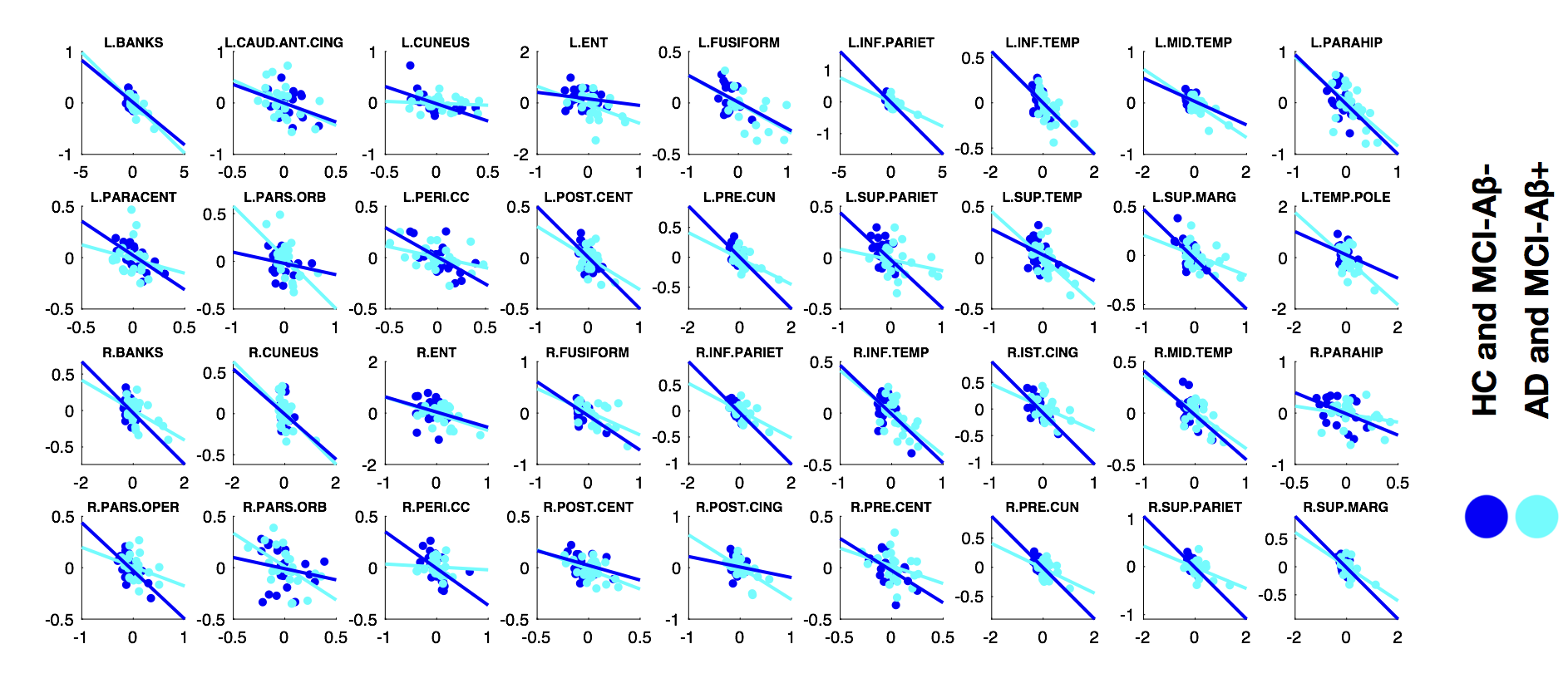
*

**Supplementary Figure 2. Scatter plots of the strongest local associations between tau burden and cortical thickness (FDR p < 0.05).** There was no significant interaction of Aβ group on the correlations. Blue: Healthy controls and MCI-Aβ -, Cyan: MCI-Aβ+ and AD.


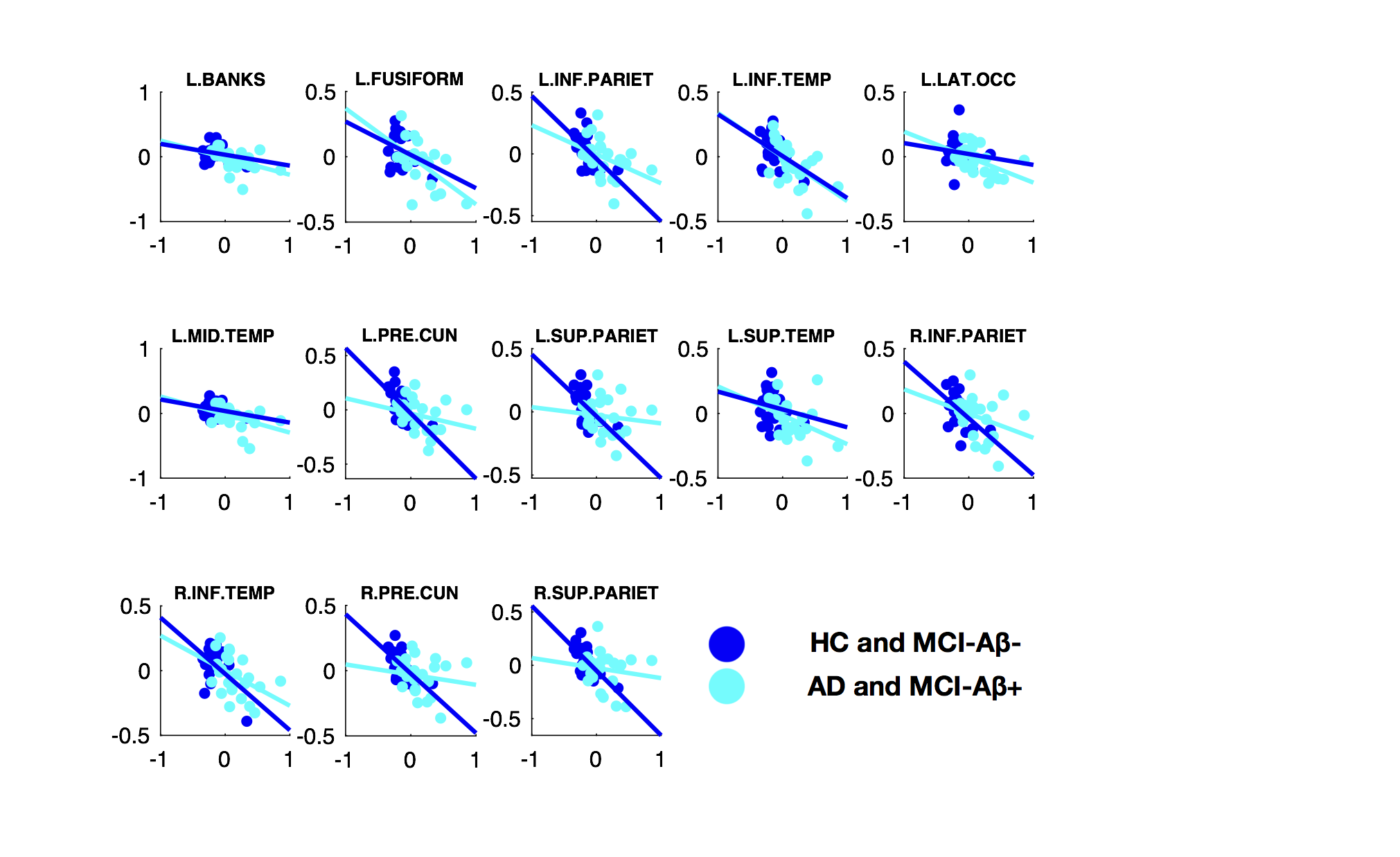


**Supplementary Figure 3. Delineating the local and distributed associations of tau in inferior temporal cortex and cortical thinning.** Scatter plots of the strongest local associations between focal tau burden in the inferior temporal ROI and whole-brain cortical thickness (FDR adjusted p < 0.05). There was no significant interaction of Aβ group on the correlations. Blue: Healthy controls and MCI-Aβ -, Cyan: MCI-Aβ+ and AD.
